# Production and Evaluation of Kleija-Like Biscuits Formulated with Sprouted Mung Beans

**DOI:** 10.3390/foods14091571

**Published:** 2025-04-29

**Authors:** Hassan Barakat, Raghad M. Alhomaid, Raya Algonaiman

**Affiliations:** Department of Food Science and Human Nutrition, College of Agriculture and Food, Qassim University, Buraydah 51452, Saudi Arabia; r.alhomaid@qu.edu.sa (R.M.A.); 411200162@qu.edu.sa (R.A.)

**Keywords:** Kleija, biscuit, mung bean, nutrition, food supply, dietary intake

## Abstract

Recently, significant interest has been shown in developing enhanced, nutrient-dense snacks. This study aimed to develop a Kleija-like biscuit, a traditional Saudi Arabian product, enhanced with sprouted mung bean flour (SMBF) to improve its nutritional profile while maintaining key sensory characteristics. The biscuits were formulated by partially replacing wheat flour with varying proportions of SMBF (10–40%), followed by a comprehensive evaluation of the biscuits’ characteristics. The results showed a significant increase in protein content, with marked enhancement in the amino acid profile. Essential amino acids, such as lysine and histidine, attained a biological value estimated at approximately 55%. Additionally, the essential amino acid index for samples containing 30–40% SMBF was reported to be around 60%. Furthermore, enhanced levels of phenolic acids and flavonoids were observed in biscuits with high SMBF content, with total flavonoids and carotenoids rising over 50%. Consequently, antioxidant activity improved markedly, with increases ranging from 20% to 45%. Furthermore, the glycemic response of these biscuits demonstrated a notable reduction. Sensory evaluations indicated high consumer acceptance, particularly in taste, texture, and overall appeal. However, the inclusion of 40% SMBF resulted in lower acceptance, suggesting that while higher SMBF levels enhance the nutritional profile, they may adversely affect sensory qualities if not balanced. Future research should focus on optimizing SMBF levels and investigating ingredient combinations to enhance flavor while satisfying consumer health and taste preferences for commercial scalability. In conclusion, incorporating SMBF into biscuit production presents significant potential for developing nutrient-dense snacks for individuals combating obesity and associated chronic diseases.

## 1. Introduction

In recent years, Saudi Arabia has experienced a significant and rapid nutritional transition characterized by a notable increase in the intake of foods high in sugars and starches [1]. This shift aligns with broader global trends, where many countries are witnessing similar changes in dietary patterns and lifestyle choices. Such transformations are closely linked to the public health and nutritional status of the population, contributing to a dramatic rise in obesity and associated chronic diseases, including type 2 diabetes and cardiovascular disease [2]. This evolving dietary landscape raises concerns about long-term health outcomes and underscores the need to promote healthier eating habits.

One of the most iconic traditional snacks in Saudi Arabian cuisine is Kleija, which originates from the Qassim region. This unique baked pastry features a balanced combination of sweet and sour flavors, with a crisp exterior and a soft, chewy filling made with a sugary mixture. The preparation of Kleija typically involves crafting a dough made from wheat flour, sugar, milk, and fat, which is then shaped into a classic round form. The filling’s flavor profile features unique ingredients like black lemon and a blend of spices, including cardamom and cinnamon. Furthermore, an annual Kleija celebration is held in the Qassim region, underscoring its significance not only as a cherished traditional product but also as a vital symbol of cultural heritage [3,4]. Therefore, developing a baked product inspired by Kleija, enriched with bioactive compounds, presents a promising opportunity to create a product that is both appealing and beneficial. In addition, bakery products, particularly biscuits, are widely recognized as convenient snacks due to their affordability, accessibility, diverse range of flavors, and extended shelf life. These attributes significantly enhance their acceptability across various demographic groups, making them a favored choice among consumers of all ages [5,6]. The fortification of bakery products with functional and innovative ingredients has become a prevalent strategy aimed at enhancing their health benefits and nutritional profiles. This approach not only addresses consumer preferences for healthier alternatives but also fosters an enhancement in dietary intake across various age groups, contributing to the replacement of less nutritious, sugary snacks with healthier options [7,8]. Researchers worldwide have highlighted the considerable potential for developing enriched and enhanced biscuit products by incorporating innovative ingredients that are not typically employed in conventional biscuit production. Ingredients such as quinoa flour [5], apple powder [7], date powder [8], and sunflower flour [6] each contribute distinct nutritional and sensory qualities to the final product. Recent innovative research has explored the inclusion of legumes in baked goods, demonstrating their potential benefits for nutrition and product quality. Legumes hold a significant importance in human nutrition, primarily due to their substantial protein content, which typically ranges from 20% to 50% [9,10].

The selection process among the diverse array of legumes presents a considerable challenge. Mung beans (*Vigna radiata*) are acknowledged for their excellent nutritional profile among the wide variety of legumes. They exhibit high protein content, ranging from 14 to 33 g per 100 g (dry matter) [11], and are notably rich in essential minerals like iron, with concentrations between 5.9 and 7.6 mg per 100 g [12,13]. They also contain various vitamins and biologically active compounds [9], making them a valuable addition to health-focused diets. Moreover, mung beans demonstrate a superior digestibility profile relative to various other legumes, including chickpeas and kidney beans [14,15]. While mung beans contain anti-nutritional factors such as phytic acid, tannins, and polyphenols, their concentrations are generally lower or more manageable in comparison to those found in other legumes [11]. In addition to their nutritional aspects, mung beans represent a sustainable agricultural crop due to their environmentally friendly characteristics. They require less water and fertilizer compared to many other leguminous plants, making them a valuable option for sustainable farming practices [16]. This combination of sustainability, digestibility, and nutritional profile underscores the status of mung beans as a valuable option within the legume category, offering benefits that extend to both nutritional and ecological contexts. Research exploring the incorporation of mung beans into biscuit production has yielded encouraging results, particularly in terms of nutritional enhancement [13,17]. On the other hand, research has demonstrated that the application of various processing techniques can markedly affect the production of nutrient-dense products. Germination, in particular, is a well-established technique recognized for its ability to enhance the nutritional profile of raw materials. Numerous studies have shown that this technique improves the bioavailability of amino acids while also increasing the concentrations of phytonutrients and associated antioxidants [18,19]. These enhancements not only increase the overall nutritional value of germinated products but also strengthen their potential health benefits in relation to diseases such as diabetes and cardiovascular conditions [20]. In light of this context, the present study aimed to investigate the incorporation of sprouted mung bean flour into Kleija-inspired biscuits.

## 2. Materials and Methods

### 2.1. Ingredients

Whole wheat flour (First Mills Co., Buraydah, Qassim, Saudi Arabia), green mung beans (locally sourced), old-fashioned rolled oats flour (manufactured by Hanna, Federal Oats Mills, Butterworth, Malaysia), cow butter, Sucrose fine powder, fresh egg, edible salt, citric acid, ground cinnamon, ground cardamom, ground black lemon, and spices were purchased from the local market of Buraydah (Qassim, Saudi Arabia).

### 2.2. Sprouting Process of Green Mung Bean Seeds

Sprouted mung bean flour (SMBF) was prepared following a previously described method [21]. Prior to the sprouting procedure, dust and fragmented seeds were meticulously removed, and the seeds were subjected to comprehensive cleaning. The seeds were sprouted promptly and harvested five days post-cleaning operation. The sprouting procedure was conducted at a controlled temperature of 20 ± 1 °C and a relative humidity of 90–93% utilizing the sprouting machine (Easygreen, Model: EGL 50, Saugeen Shores, ON, Canada). Then, sprouts were frozen overnight at −80 ± 1 °C and freeze-dried for 72 h at −52 °C and 0.060 mbar (CHRIST, Alpha 1–2 LD plus, Osterode, Germany). A fine powder was produced by milling freeze-dried sprouts in a small mill (Thomas Wiley, Philadelphia, PA, USA) and sieving the product through a 60-mesh sieve. The powder was subsequently stored in opaque containers at 4 ± 1 °C until utilized for chemical analysis or biscuit formulation.

### 2.3. Formulation and Processing of Biscuit Samples

Different Kleija-like biscuit formulas were manufactured at the Food Factory of Agriculture and Food College, Qassim University. Five biscuit formulas were carried out using the ingredients presented in Table 1. All ingredients were prepared at room temperature; then the sucrose fine powder was added to the cow butter and mixed for 5 min in a mixer (Kenwood Chef XL Food Processor OWKVL4230S, De’Longhi Deutschland GmbH, Neuss, Germany) at speed 4 to achieve a homogeneous creamy texture. For each formula, 35 g of whipped egg with 2 mL liquid vanilla were added; then, the mixing process was extended for 3 min. Subsequently, all dry ingredients, including whole wheat flour, sprouted mung bean flour, edible salt, spice mix, citric acid, and baking powder, were sieved and gradually added during kneading. The kneading process was continued at speed 4 until a homogeneous dough was formed.

**Figure 1:**
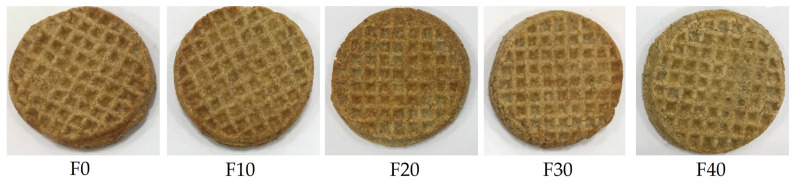
Kleija-like biscuit formulas baked at 180 °C for 15 min. F0–F40: formulations of biscuits, see Table 1.

The dough was sheeted using a rolling pin to a thickness of 6 mm with the help of an aluminum platform and a frame, then cut using a circular frame to flatten round dough pieces. The flattened round dough pieces were shaped using a unique wood frame by pressing the dough into a 4 mm diameter, brushed with an appropriate amount of whipped egg in milk mix, then baked on an aluminum tray at 180 °C for 15 min (Figure 1). The biscuits were cooled for 30 min and stored in air-tight tins for 24 h before further analysis.

### 2.4. Chemical Analysis

#### 2.4.1. Proximate Composition and Mineral Content

The prepared biscuit samples were subjected to chemical analysis and caloric estimation according to the standard methods of AOAC [22]. The content of minerals was analyzed using atomic absorption spectroscopy following the protocols of AOAC, and a colorimetric method was employed for phosphorus, as mentioned by Borah et al. [23]. The β-glucan content in the prepared biscuit samples was investigated utilizing a mixed linkage (1–3, 1–4) β-glucan test kit manufactured by Megazyme by Neogen (Wicklow, Ireland) in accordance with the manufacturer’s methodology, as detailed by Algonaiman and Alharbi [24].

#### 2.4.2. Analysis of Phytochemical Content and Antioxidant Capacity

Formulated KLBs have been extracted 3 times using 70% methanol as a solvent and centrifuged at 10,000× *g* for 10 min. Then, the clear supernatant has been collected for colorimetric analysis. According to Nsimba et al. [25], the total phenolic content of the formulated biscuit samples’ extracts was determined using the Folin–Ciocalteu reagent. The total flavonoid (TF) content was determined in the samples’ extracts according to the method described by Mohdaly et al. [26]. Total carotenoids were determined according to Yuan et al. [27]. The DPPH radical scavenging activity of the samples was measured according to Nsimba et al. [25]. The ABTS (2,2′-azino-bis(3-ethylbenzothiazoline-6-sulphonic acid) radical cation was measured by the adapted method of Lu et al. [28].

#### 2.4.3. Quantification of Phenolic Compounds

According to Schneider [29], the phenolics in KBs incorporating GMB were analyzed by the HPLC system Agilent1260 Infinity Series (Agilent Technologies, Palo Alto, CA, USA), which is equipped with a Quaternary pump, an autosampler, and a Variable Wavelength Detector (VWD, Hewlett-Packard 1050) set at 280 nm. The system uses a column akinetex^®^ 1.7 µm EVO C150 mm × 4.6 mm (Phenomenex, Torrance, CA, USA) and is operated at 30 °C. The separation was achieved using a ternary linear elution gradient with (A) HPLC grade water of 0.1% Trifluoroacetic acid (TFA), (B) acetonitrile, and (C) HPLC grade methanol. The gradient elution started with 94:3:3 (*v*/*v*) for phenolics and 98:1:1 (*v*/*v*) for flavonoids; 20 µL of the sample extract was automatically injected; the flow rate was set at 1 mL min^−1^; the separation was carried out at 30 °C, and the environmental temperature was 20 °C. Peaks of the phenolic compound were quantified as mg kg^−1^ using external calibration curves of relevant standard solutions.

#### 2.4.4. Determination of Amino Acid Profile

The amino acids profile of different KBs incorporating GMB were determined using a Sykam amino acid analyzer (Sykam GmbH, Eresing, Germany) equipped with solvent delivery system S 2100 (Quaternary pump), autosampler S 5200, amino acid reaction module S4300 with built-in dual filter photometer between 440 and 570 nm with constant signal output and signal summary option, and refrigerated reagent organizer S 4130.

##### Instrument Parameters

The instrument was equipped and stetted with column LCA K06/Na; the mobile phase was buffer A (11.8 g Tri-sodium citrate dihydrate, 6 g citric acid, 65 mL methanol, 6.5 mL HCL 32%, and 0.5 phenol per L, pH 3.45 and Normality 0.12) and buffer B (19.6 g Tri-sodium citrate dihydrate, 3.1 NAOH, and 5 g boric acid per L, pH 10.85 and Normality 0.2); mode of elution was gradient; the flow rate was mL min^−1^; the gradient temperature was adjusted from 57 to 74 °C, and the used wavelengths were 440 and 570 nm.

##### Standard and Sample Preparations

Standard Stock solution type H (Sykam CAT number S000029), which contains 18 amino acids (aspartic acid, threonine, serine, glutamic acid, proline, glycine, alanine, cystine, valine, methionine, isoleucine, leucine, tyrosine, phenylalanine, histidine, lysine, ammonia, arginine) at concentrations of 2.5 µmol mL^−1^ was applied for all amino acids, except cysteine, which was used as 1.25 µmol mL^−1^ in 0.1 A total of 60 µL of HCL containing phenol (0.1% *w*/*v* as preservative) was diluted in 1.5 mL vial with HPLC water, filtered using 0.22 µm syringe filter; then, 100 µL was injected. For sample preparation, around 1 g (4 decimals accurately recorded for calculations) of each sample was mixed with 20 mL 6N HCL in a pressure vessel and then incubated in an oven for 16 h at 110 °C. After the incubation, the sample was filtered on Whatman No. 1 filter paper, evaporated on a rotary evaporator, and dissolved completely in 100 mL HPLC Water. From this solution, 1 mL was diluted in 10 mL HPLC water, filtered using a 0.22 µm syringe filter, and then, 100 µL was injected. Both the sample and the single-level standard are analyzed under identical conditions. The concentration of each amino acid in the sample is calculated based on the relative response factor derived from the standard, according to Cohen et al. [30]. The predicted biological value (BV) and amino acids score depends on WHO, 2007 [31] and has been calculated as cited by Chavan et al. [32] using indicated equations as follows:

$$BV=10^{2.15}\;\times \;q_{Lys}^{\;0.41}\;\times \;q_{Phe+Tys}^{\;0.60}\;\times \;q_{Met+Cys}^{\;0.77}\;\times \;q_{Thr}^{\;2.4\;}\;\times \;q_{Trp}^{\;0.21}$$

where

***q*** *=* $\frac{{ai\;}_{Sample}}{{ai\;}_{Reference\;}}$ for *ai* samples ≤ *ai* reference;

***q*** *=* $\frac{{ai\;}_{Reference}}{{ai\;}_{Sample\;}}$ for *ai* samples ≥ *ai* reference;

*ai* = mg of the amino acid per g of total essential amino acid;

$$Amino\;acid\;score=\frac{mg\;of\;the\;amino\;acid\;per\;test\;protein}{mg\;of\;amino\;acid\;per\;g\;of\;FAO/WHO\;standard\;pattern}\times 100$$

#### 2.4.5. In Vitro Glycemic Index (GI) and Glycemic Load (GL)

The GI was estimated using the method of Aribas et al. [33], which was modified by Alwohaibi et al. [34]. Briefly, they were digested using pepsin (0.117 g mL^−1^, Sigma, P3000, Kawasaki, Japan), pancreatin (0.243 g, Sigma, P3000), and amyloglucosidase from *Aspergillus niger* (260 U/mL, Sigma). The enzyme activity of the samples taken promptly was stopped using absolute ethanol and then centrifuged. Glucose content was measured with a glucose determination kit, GOD-PAP (Human Co., Wiesbaden, Germany) at 500 nm wavelength. Absorbance values were plotted against time to draw the hydrolysis curve, and GI, HI, and GL were calculated using the relevant equations.

#### 2.4.6. Degree of Proteolysis

The water-soluble extract (WSE) of all FCMs was first prepared according to Shori et al. [35]. Briefly, 10 g of FCM were mixed with 40 mL d.H_2_O and homogenized, kept at 40 °C for 1 h, and then centrifuged at 10,000× *g* for 30 min. The clear supernatant WSE was collected and stored at −80 °C until analysis. A previously reported O-phthaldialdehyde (OPA) method was used to measure the total free amino acids (mmol) of the FCM samples [36].

### 2.5. Physicochemical Properties

#### 2.5.1. Amylograph

Amylograph is primarily used for assessing starch’s gelatinization and pasting behavior, providing detailed viscosity profiles regarding α-enzymatic activity, and is crucial for understanding starch functionality in food products. The Brabender Amylograph characteristics of composite flour incorporating SMBF used for producing Kleija-like biscuits were determined using the standardized method (AACC 22-10.01) of AACC [37]. Concisely, three parameters are recorded: gelatinization temperature (°C), peak viscosity (AU), and the temperature at peak viscosity (°C), which are presented in Figure 3.

#### 2.5.2. Measurement of Instrumental Color

The color parameters of the prepared biscuit samples were determined using a ColorFlex colorimeter (ColorFlex, Reston, VA, USA). The data were recorded in triplicate for the measurement of lightness (L*), redness (a*), and yellowness (b*). According to the International Commission for Color Measurement (CIE) system, the L* values range from 0 to 100, representing black to white. The a* values range from +60 to −60, representing red to green, and the b* values range from +60 to −60, representing yellow to blue. Further calculations were performed to determine the hue angle (H°), chroma value (C*), color changes (∆E), and browning index (BI) using the specified formulas described by Lavelli et al. [38]. The H° represents the color’s position on the color wheel; C* indicates the color intensity; ∆E measures the color difference. At the same time, the BI quantifies browning or color development in food products, particularly in baked goods, indicating the extent of the Maillard reaction [39].

### 2.6. Sensory Evaluation

A well-trained panel of 14 qualified and experienced arbitrators conducted the sensory evaluation of the prepared biscuit samples. They evaluated multiple selected sensory attributes, including appearance (20), taste (20), external color (15), internal color (15), texture (15), and aroma (15), using specified scores and overall acceptability (100) as a summation of all of the sensory attributes. Samples were randomly picked from each of the three pilot plant batches tempered for 30 min at 25 °C in the sensory analysis room. The consumption ratio was half a whole biscuit from each reference, maintained at a temperature of 25 °C. Water was provided to the evaluators for oral cleansing between the various samples. The samples were evaluated without replication. To assess the reliability of the results, the control biscuit was incorporated twice into the evaluations, randomly interspersed among other samples. The obtained results were statistically analyzed and expressed using the method described by San José et al. [40] after appropriate modification.

### 2.7. Statistical Analysis

The data were statistically analyzed using SPSS software package version 25 (IBM Corp., released in 2013, New York, NY, USA). One-way analysis of variance (ANOVA) was applied, assuming a complete randomization design for the data. The Tukey test was used for multiple comparisons, and a significance level of ≤0.05 was applied. The results were expressed as mean ± standard deviation (SD) or standard error of the mean (SE).

## 3. Results

### 3.1. Proximate Composition

The proximate composition of the prepared biscuit samples showed multiple changes as a result of incorporating SMBF (Table 2). The moisture content increased with higher percentages of SMBF, rising by 45–121% from F0 to F30. However, F40 exhibited a moisture content 42% lower than F30. The fat content showed a minimum decrease ranging from 0.4% to 3.4%. Meanwhile, the carbohydrate content showed a slight decrease, ranging from 2% to 7%, with an increase in SMBF content. This minimum reduction in fat and carbohydrate content subsequently resulted in a slight decline in energy consumption by 0.3–1%. Consistently, the crude fiber content showed a modest reduction, ranging from 5–12% compared to F0. In contrast, the soluble fiber, primary β-glucan, showed a notable increase with the increase in SMBF content. As presented in Table 2, the samples exhibited β-glucan content ranging from approximately 293 to 325 mg 100 g^−1^, compared to that observed in the control sample with 268 mg 100 g^−1^. Furthermore, the incorporation of SMBF resulted in a significant, dose-dependent increase in protein content, with an enhancement of up to 47% compared to the control. Lastly, the analyzed ash content considerably increased with the increase in SMBF. The higher incorporation percentage showed an increase in the ash content by 18% compared to that observed in the control sample.

### 3.2. Mineral Content

The mineral content of the prepared Kleija-like biscuits was measured to assess the effect of SMBF incorporation (Table 3). The control sample recorded acceptable amounts of different elements, including calcium, sodium, potassium, phosphorus, magnesium, manganese, iron, zinc, copper, and selenium. The formulated biscuit samples showed an interesting rise in most of these elements. An increase of 7–28% was observed in calcium content, with F30 and F40 recording the highest rise (82 and ~86 mg 100 g^−1^, respectively, vs. ~67 mg 100 g^−1^ in F0). The potassium content increased significantly by 69–80% in F30 and F40, rising from 271 mg 100 g^−1^ in F0 to 459 mg in F30 and 500 mg in F40. Phosphorus and magnesium also showed a consistent increase of 14–17% in both F30 and F40. Similarly, the content of iron showed a marked increase by 20–25% in both F30 and F40 (~53 and ~55 mg 100 g^−1^, respectively, vs. ~44 mg 100 g^−1^ in F0). The other measured elements, including sodium, copper, and zinc, showed a rise across all of the tested samples by 20–80%, 21–86%, and 2–8%, respectively. The results further showed a slight decline in the content of other elements, including manganese and selenium (Table 3). These two elements declined across all tested samples by 3–11% and 6–22%, respectively.

### 3.3. Phytochemicals and Antioxidant Capacity

The phytochemical content measured in the prepared samples showed considerable changes as a result of incorporating the SMBF (Table 4). An increase of 16% in total phenolic content was observed in F40, whereas the levels in F10 to F30 exhibited a minimal increase in the range of 2% to 7%. In contrast, total flavonoids and carotenoids demonstrated a significant increase exceeding 50%. The total flavonoid content exhibited a gradual increase from 129.66 ± 0.74 mg QE 100 g^−1^ in F0 to 166.57 ± 1.45, 179.89 ± 2.69, 200.2 ± 2.27, and 212.63 ± 4.30 mg QE 100 g^−1^ in F10, F20, F30, and F40, respectively. Whereas the total carotenoids increased from 22.02 ± 1.11 mg GAE 100 g^−1^ in F0 to 28.3 ± 1.32, 30.6 ± 1.05, 34.0 ± 1.04, and 36.2 ± 0.87 mg GAE 100 g^−1^ in F10, F20, F30, and F40, respectively. These results unequivocally demonstrate that the highest quantity is observed in F30 and F40, indicating that a higher content of SMBF is associated with a higher increase in phytochemical content. Furthermore, the samples underwent antioxidant activity measurements, as shown in Table 4. The addition of SMBF resulted in significant antioxidant activity based on both DPPH and ABTS measuring assays. The activity increased in a dose-dependent manner by 24%, 29%, 39%, and 45% in F10, F20, F30, and F40, respectively.

### 3.4. Phenolics Fractionations

The phenolic fractionation of the prepared Kleija-like biscuit samples, as well as the SMBF, was analyzed by the HPLC system, as shown in Table 5. The analysis identified eight distinct phenolic acids present in the SMBF sample. Ferulic acid and benzoic acid recorded the highest levels of approximately 252 and 159 mg kg^−1^, respectively. Followed by caffeic, ellagic, and syringic acids found in moderate amounts in the range of 76–78 mg kg^−1^. The remaining phenolic acids, chlorogenic, *O*-coumaric, and *p*-coumaric acids, were found in much lower concentrations. The analysis further identified six flavonoid compounds, with kaempferol and naringenin exhibiting the highest concentrations of approximately 280–284 mg kg^−1^. These were followed by myricetin and rutin, detected at levels of 113 and 95 mg kg^−1^, respectively. Quercetin and resveratrol were also identified, but at lower concentrations of 54 and 37 mg kg^−1^, respectively. The analyzed formulated biscuit samples displayed dynamic changes in their phenolic content. As shown in Table 5, the highest detection levels were noted in catechol, with concentrations ranging from approximately 4880 to 12,414 mg kg^−1^ recorded in F0 to F40. Notably, these concentrations showed a dramatic increase with the increase in the ratios of SMBF. Phenolic acids also showed considerable variations in the samples, with gallic acid followed by *O*-coumaric acid, ferulic acid, and benzoic acid reaching 103, 91, 50, and 19 mg kg^−1^, respectively. Notably, these concentrations were exclusively detected in the F40 sample. Other phenolic acids, such as *p*-hydroxybenzoic acid, were detected in all samples, ranging from 57 to 95.5 mg kg^−1^. In contrast, phenolic acids such as *p*-coumaric acid and syringic acid were only identified in the control sample at lower concentrations. Vanillic acid was also identified in all samples with varying concentrations, with the control sample exhibiting 641 mg kg^−1^, while the F20 sample reached 1044 mg kg^−1^. In contrast, the F40 sample displayed a significant decrease to 398 mg kg^−1^. Furthermore, the analysis of flavonoid compounds in the formulated samples revealed the presence of five distinct flavonoids, as summarized in Table 5. Notably, the F40 sample exhibited elevated kaempferol, apigenin, and myricetin concentrations, quantified at 335, 287, and 125 mg kg^−1^, respectively. In contrast, the F20 samples demonstrated lower concentrations of these three flavonoids, with respective amounts of approximately 19, 21, and 112 mg kg^−1^, respectively. In addition, naringenin was identified in the F40 sample at a lower concentration of 25 mg kg^−1^. Quercetin was also detected in both F20 and F40 samples, with concentrations ranging from 21 to 33 mg kg^−1^. In comparison, the control sample displayed flavonoids limited to quercetin and rutin, with quercetin quantified at a higher concentration of ~273 mg kg^−1^, while rutin was present at a lower concentration of ~26 mg kg^−1^. Nonetheless, these findings indicate that samples containing higher SMBF ratios exhibited excellent phenolic compound detection rates.

### 3.5. Glycemic Index (GI) and Glycemic Load (GL)

The glucose release intensity of the prepared Kleija-like biscuit samples was assessed in vitro using the parameters HI, GI, and GL (Table 6). This analysis revealed a gradual reduction in the HI corresponding to the decreased content of whole wheat flour compared to the control sample (F0). Lower HI was notably observed in F40 compared to that in the control sample. Similarly, the GI values showed a gradual reduction, with the F40 sample demonstrating the most considerable decrease compared to the control sample. Notably, both the F30 and F40 samples demonstrated GI values comparable to those seen in the SMBF sample, with values of 40.02 ± 0.03 and 40.00 ± 0.02, respectively, versus the control value of 40.02 ± 0.03. Moreover, the GL exhibited a notable reduction, declining by 5–6% in both F30 and F40.

### 3.6. Amino Acid Profile

The amino acid profile of the prepared Kleija-like biscuit samples was thoroughly analyzed (Table 7). The incorporation of SMBF led to a significant increase in most of the essential amino acids across all formulated samples. Lysine exhibited a notable increase in mean concentration, rising from ~27 mg g^−1^ protein in the F0 sample to 41 mg g^−1^ protein in both the F30 and F40 samples. In addition, both the F30 and F40 samples exhibited a considerable increase in mean concentrations of histidine and isoleucine, ranging from 20% to 45%. Other essential amino acids, including valine, threonine, phenylalanine, and leucine, exhibited a modest increase ranging from 2% to 11% across all formulated samples. However, methionine and cystine displayed a mild decrease, ranging from 6% to 9%. In general, the mean concentration of the total essential amino acids demonstrated a considerable increase, rising from 343 mg g^−1^ of protein in the F0 sample to 385 mg g^−1^ of protein in the F40 sample. The results also demonstrated a notable increase in the ratio of essential amino acids to total amino acids, rising from 0.34 in F0 to 0.39 in F40. This indicates that 39% of the total amino acids are essential amino acids, based on the estimated mean concentrations. Furthermore, the analysis of non-essential amino acids indicated a notable increase in all formulated samples (Table 7). Aspartic acid demonstrated a notable increase in mean concentrations, rising from 63 mg g^−1^ of protein in F0 to 88 mg g^−1^ of protein in F40. Arginine mean concentrations increased significantly from approximately 42 mg g^−1^ of protein in F0 samples to 56 mg g^−1^ in F40 samples. The observed increase in both aspartic acid and arginine concentrations represents an estimated increase of 40% and 35%, respectively. Other non-essential amino acids, particularly tyrosine and alanine, exhibited a minor increase across the formulated samples, ranging from 1% to 5%. In contrast, non-essential amino acids, such as serine, glutamic acid, proline, and glycine, demonstrated a slight decrease in their mean concentrations. Subsequently, the mean concentrations of total non-essential amino acids decreased from ~657 mg g^−1^ of protein in F0 to ~615 mg g^−1^ of protein in F40.

In the context of a comprehensive analysis of amino acids, their classifications were explored, as presented in Table 8. The branch-chain amino acids, which comprise the essential amino acids leucine, isoleucine, and valine, exhibited a notable increase in their concentrations, rising from ~134 mg g^−1^ of protein in the F0 sample to 153 mg g^−1^ of protein in the F40 sample. Similarly, basic amino acids, including lysine, arginine, and histidine, increased from ~109 mg g^−1^ protein in the F0 sample to ~146 mg g^−1^ protein in the F40 sample. Other amino acids, including both acidic and aromatic types, exhibited modest increases of 1% and 4%, respectively, in comparison to the levels observed in the control sample. Whereas polar amino acids, encompassing serine, threonine, asparagine, glutamine, and cysteine, exhibited no notable variations among the formulated samples. In contrast, the concentration of conditional amino acids demonstrated a remarkable decline of 13%, consistent with the reductions observed in Table 7. This decrease encompasses several amino acids, including arginine, cysteine, glutamine, tyrosine, and glycine. Similarly, the hydrophobic amino acids, which include alanine, valine, leucine, isoleucine, phenylalanine, and methionine, exhibited a reduction of 10%. Nonetheless, the calculated biological value percentage of the amino acids in the F20 to F40 samples was approximately 55%. In contrast, the control sample demonstrated a biological value of 45%. Furthermore, the essential amino acid index (EAAI), a vital metric for evaluating the quality of protein sources based on their composition of essential amino acids, exhibited values ranging from 57 to 59 in the samples F20 to F40. This index reflects the extent to which a protein source fulfills the body’s requirements for these vital amino acids. In this context, Table 8 also presents the requirement index for various age groups. The infants’ requirement index demonstrated a considerable increase, rising from approximately 111 to about 127 in samples F0 to F40. Similarly, the requirement indices for preschool children and school-aged children demonstrated consistent increases, rising from 120 to 138 and from 131 to 151, respectively, in the transition from F0 to F40. Additionally, the adult requirement index demonstrated a similar upward trend, increasing from 138 in F0 to 158 in F40. These findings underscore the substantial changes in nutrient requirements across the different age groups, reflecting a percentage increase of 15% when compared to the control sample.

### 3.7. Degree of Proteolysis

**Figure 2:**
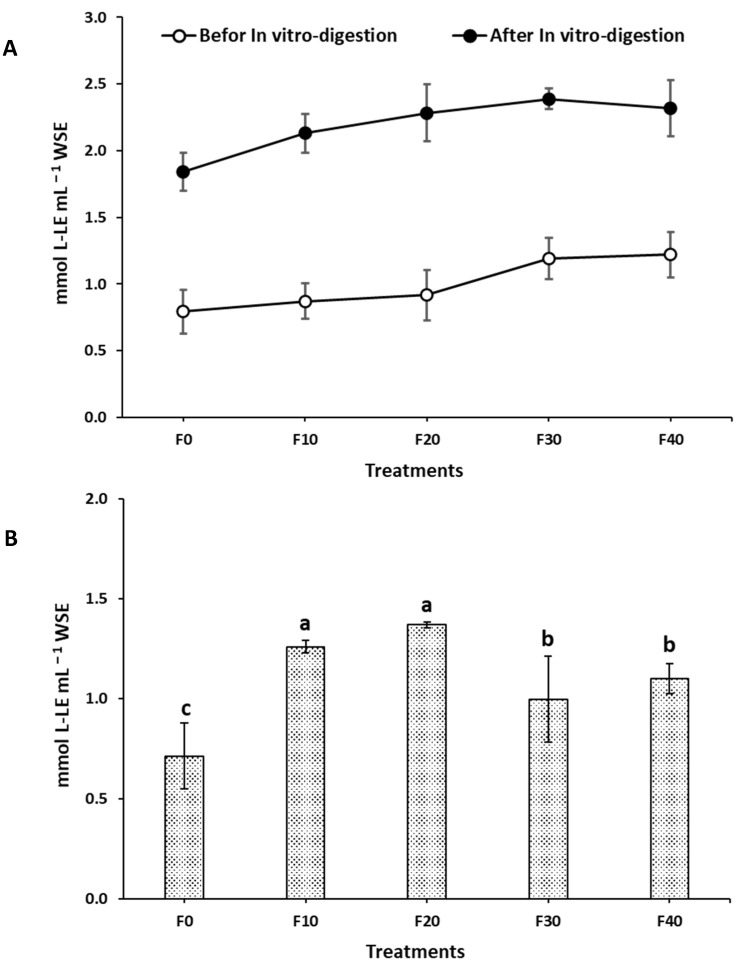
Degree of proteolysis (mmol L-LE mL^−1^) for different Kleija-like biscuits incorporated with SMBF. (F0–F40): formulations, see Table 1; ^a,b,c^: bars sharing same letters are not statistically different (*p* < 0.05).

A water extract of the prepared Kleija-like biscuits was utilized to assess the degree of in vitro proteolysis, defined as the extent to which proteins are hydrolyzed into smaller peptides and amino acids by the action of proteolytic enzymes. As illustrated in Figure 2A, a remarkably heightened degree of proteolysis was observed following the in vitro digestion when compared to pre-digestion levels. These results indicate an effective degradation of proteins into smaller peptides and amino acids, highlighting the efficiency of the digestive process. Notably, a positive correlation was identified between the proportion of SMBF and the extent of proteolysis; as the proportion of SMBF increased, there was a corresponding rise in the extent of protein degradation. Furthermore, Figure 2B illustrates a significant statistical difference in the degree of proteolysis when compared to the control sample. Both F20 and F30 exhibited markedly higher levels of protein degradation relative to F0. Significantly increased protein degradation was also observed in both F30 and F40 compared to F0; however, their degradation levels were notably lower than those observed for F20 and F30.

### 3.8. Amylograph

**Figure 3:**
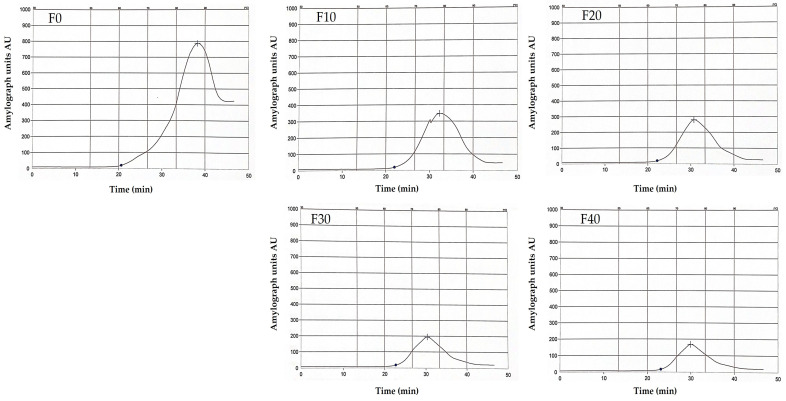
Amylogram of different composite flour incorporated with sprouted mung bean flour utilized in producing Kleija-like biscuits. (F0–F40): formulations, see Table 1.

The gelatinization properties of the prepared Kleija-like biscuit samples were measured utilizing the Amylograph instrument. As presented in Table 9, the gelatinization temperature, which refers to the temperature at which starch granules absorb water and swell, varied between 60.9 °C for the control sample (F0) and 64.6 °C for sample F40. F0 demonstrated the lowest gelatinization temperature, indicating a quicker formation of gel compared to F40, which required approximately 65 °C to achieve gel formation. In addition, the peak viscosity temperature, the point at which the starch paste reaches its maximum viscosity, was remarkably higher in the F0 sample (87.4 °C) compared to that observed in the F40 sample (74.9 °C). The maximum peak viscosity also demonstrated a substantial difference between the two samples, with the F0 sample exhibiting a viscosity of 788 AU versus only 168 AU for the F40 sample. Furthermore, the analysis of starch breakdown over time, as shown in Figure 3, reveals distinct responses among the tested samples. The pronounced peak observed in F0, occurring between 20 to 40 min, signifies a rapid initial degradation of starch. In contrast, smaller peaks were observed in F10 and F20, suggesting a decline in the rate of starch breakdown during this period. F30 and F40 further displayed the lowest peaks, indicating minimal starch degradation at these time points. These observations suggest that samples with lower concentrations of whole wheat flour relative to the control sample exhibited a corresponding decrease in starch content, which, in turn, resulted in reduced viscosity.

### 3.9. Color Measurements

The prepared Kleija-like biscuit formulations underwent color measurement to evaluate the impact of incorporating SMBF on the color appearance. As presented in Table 10, the samples exhibited significant changes in their color based on the significant differences observed in the values of the color change indicator (∆E). Lighter colors were observed with increased SMBF concentrations. From F10 to F40, the lightness indicator demonstrated a dose-dependent increase ranging from 1% to 9%. This rise in lightness was accompanied by a corresponding decrease in the browning index, with reductions of 3%, 15%, 18%, and 26% observed in F10, F20, F30, and F40, respectively. In addition, a significant reduction of over 50% in color redness was observed in F40. The results also showed a decline in color intensity, with decreases of 8% and 10% noted in F30 and F40, respectively.

### 3.10. Sensory Evaluation

The prepared Kleija-like biscuit samples were subjected to sensory evaluation to assess their taste, aroma, texture, appearance, and overall palatability (Table 11). The control samples demonstrated marginally higher scores in terms of appearance compared to those formulated with SMBF. However, these differences were not statistically significant, suggesting that the appearance of the formulated biscuits remained acceptably comparable to that of the control samples. In terms of texture, the majority of the prepared samples exhibited favorable results; however, those incorporating a higher proportion of SMBF (40%) yielded less favorable texture scores. Additionally, there was no significant difference was observed in color between the prepared samples and the control, for both the outer cover and the inner pulp colors. Furthermore, evaluating the taste profiles showed that samples containing higher proportions of SMBF received notably lower taste scores. Specifically, the F40 sample exhibited a significantly diminished score compared to the other samples, highlighting the adverse impact of increased SMBF on taste perception. Concerning aroma, the control sample demonstrated superior scores compared to the formulated samples. Nonetheless, the newly developed formulations received comparable overall acceptability ratings, with those incorporating less than 40% SMBF being particularly favored by the panelists.

## 4. Discussion

Recent innovations in producing fortified or enhanced food products have garnered considerable interest. Developing well-formulated alternatives to commonly consumed foods presents significant health benefits, particularly concerning individuals dealing with obesity and related chronic diseases such as diabetes and cardiovascular conditions [7,8,41]. In the current work, Kleija-inspired biscuits were produced in five different formulations by substituting whole wheat flour with SMBF at 10%, 20%, 30%, and 40%. At these levels of incorporation, the biscuit samples exhibited significant variations in multiple parameters. The protein content showed a significant enhancement, exhibiting an approximately twofold increase compared to the control sample, with values ranging from 9% to 11.5%. In a comparable study, substituting 60% of wheat flour with mung bean flour in biscuit production achieved a protein content of 8% in the final product [17]. In contrast, the current research achieved a higher protein content of 10.6% with a significantly lower proportion of mung bean flour (30%). Similar findings were observed in baked products formulated with 15–25% mung bean flour, yielding a protein content of approximately 9% [42]. Conversely, cookies produced with a formulation consisting entirely of mung bean flour showed a protein content of 12% [13] and 15% [43]. These observed variations can rely on factors such as the diversity of varieties, analytical methods utilized, or the processing techniques employed [20]. Mung bean flour is generally recognized for its substantial protein content, with studies estimating a range between approximately 18% [17] and 25% [10,12]. Whole mung beans also exhibit a comparable protein content, estimated at 22.2% [44]. In the current work, mung beans were subjected to a germination process prior to the production of flour. The sprouting process is well-established as a highly effective method for enhancing the nutritional profile of grains and legumes by increasing the bioavailability of amino acids [19]. Consequently, flour derived from germinated seeds possesses enhanced protein bioavailability, reflecting its potential as a high-quality nutritional source. Some research has shown that protein content in sprouted mung beans escalated from 25.5% to 28% after 24 h of germination [18]. Another investigation showed increased protein content from 22% to 26% following 72 h of germination [20]. Extended germination durations of up to 96 h were further shown to increase the protein content from 26% to 31% [19]. In the current work, the germination process was conducted over a period of 120 h. The achieved enhancement in protein content is likely attributed to this extended germination duration. It has been demonstrated that sprouted seeds undergo various enzymatic processes that lead to the breakdown of stored nutrients. Key enzymes, particularly proteases, are activated, resulting in the hydrolysis of proteins into their constituent amino acids [18,45]. In the current work, an in vitro proteolysis utilizing the biscuit samples’ extracts demonstrated a significant elevation of protein degradation correlated with higher levels of SMBF. These results suggest that samples with higher levels of SMBF may exhibit a more favorable amino acid profile. The germination process has been demonstrated to substantially improve protein quality by elevating the levels of certain essential amino acids. Specifically, the concentrations of leucine, phenylalanine, and threonine in sprouted mung beans were significantly increased in correlation with the extended germination period [18]. In the current work, notable elevations in the mean concentrations of essential amino acids were observed due to incorporating SMBF. The mean concentration of lysine, recognized as the most essential amino acid often absent in wheat goods [19], was measured at 41 mg g^−1^ of protein in samples containing 30% to 40% of SMBF. Elevated mean concentrations of histidine were also observed in samples containing higher proportions of SMBF, reaching approximately 42–48 mg g^−1^ of protein. Comparable elevated levels of lysine and histidine were detected in baked cookies formulated with SMBF in comparison to those made entirely with wheat flour [9]. The prepared Kleija-like biscuits also demonstrated notable elevations in non-essential amino acids, particularly aspartic acid, and arginine, with elevations of approximately 40% and 35%, respectively. These findings align with previous research indicating substantial increases in such amino acids in mung beans subjected to a germination period of 96 h [19]. The current results indicate a significant improvement in protein quality, with the essential to total amino acids ratio increasing from 0.34 in the control sample to 0.39 in samples containing 40% SMBF, indicating that 39% of total amino acids are essential. These samples met nutrient requirements for various age groups, with a 15% increase in nutritional adequacy compared to control samples attributed to SMBF incorporation.

Furthermore, incorporating SMBF resulted in enhancements in dietary fiber content, particularly β-glucan. Achieving elevated levels of β-glucan is recognized as a practical approach to enhancing the nutritional quality of food products [24]. Higher dietary fiber intake is strongly associated with a decreased risk of obesity and chronic diseases [46]. In the current work, the β-glucan concentration varied among samples, showing increases between 5% and 28% compared to the control. This enhancement could be attributed to incorporating oat flour into the formulations [24]. However, the proportions of oat flour remained consistent across all formulated samples, indicating that the observed elevation in β-glucan levels was likely linked to SMBF inclusion. Higher levels of SMBF substitution (30–40%) led to a 20–28% increase in β-glucan content in the formulated biscuits compared to the control samples. This observation raises a concerning question, given that mung beans are not typically acknowledged as a source of β-glucan. This polysaccharide is primarily associated with grains such as oats and barley, which are well-documented for their β-glucan content [24]. A plausible explanation might be attributed to the biosynthesis of β-glucan in the mung beans. An archival study has revealed that mung beans may contain certain components associated with the synthesis of β-glucan, particularly the enzyme β-glucan synthase, which plays a crucial role in catalyzing its formation [47]. Further studies are encouraged to explore this aspect in greater depth. Furthermore, the utilization of germination techniques can influence the dietary fiber content of legumes. Studies have shown that germinated mung bean seeds exhibit enhanced dietary fiber content, with observed increases ranging from approximately 12% to 14% [20] and from 10.6% to 13% [48]. However, it is important to note that Skylas et al. [48] observed a decrease in soluble fiber, declining from 3% to 1.5% in the germinated seeds. This indicates that while germination can enhance total dietary fiber, it may reduce the proportion of soluble fibers due to the enzymatic modifications on cell wall polysaccharides [48]. A comparable reduction in soluble fiber, particularly β-glucan, was observed in foods produced with techniques involving enzymatic and microbial activities, such as fermentation [24]. In general, mung bean flour has been documented to contain moderate amounts of total dietary fiber, with reported levels ranging from approximately 6.5% [10] to 12% [20]. Some studies have indicated even higher dietary fiber content in mung bean flour, with values reaching up to 24% [12].

On the other hand, substituting wheat flour with 10–40% of SMBF demonstrated a favorable impact on glycemic response. In particular, higher substitution levels of 30–40% led to a 5–6% reduction in glycemic load compared to the control samples. Additionally, the samples exhibited a GI value of 40, significantly lower than that of various commercially available biscuits, which typically range from 49 to 68 [49]. GI is a quantitative measure that evaluates the rate at which specific foods increase blood glucose levels following consumption. Foods with a GI of 60 or higher are rapidly digested, causing rapid spikes in blood glucose levels, while those with a low GI of less than 55 are digested more slowly, resulting in gradual elevations in blood glucose [50]. The observed reduction in GI can be attributed to the decrease in wheat flour proportions, which ultimately leads to a lower carbohydrate content. A reduction of 7% in carbohydrate levels was observed; however, this modest decrease may impact the overall starch content. The results showed that the formulated biscuit samples required higher gelatinization temperatures compared to the control biscuits. This observation indicates that the gelatinization process occurred at a slower rate, presumably due to the lower starch content in the formulated biscuits. As a result, these findings imply a more gradual and consistent rise in blood glucose levels, contributing to an improved glycemic response [51]. Furthermore, it is worth noting that mung bean flour has been recognized as a good source of resistant starch [48], a type of starch that escapes digestion in the small intestine and instead undergoes fermentation in the large intestine. This fermentation process is critical in enhancing insulin sensitivity and glucose metabolism, primarily through producing short-chain fatty acids (SCFAs) such as butyrate, propionate, and acetate. These SCFAs have been shown to enhance glucose uptake and improve insulin signaling across various tissues [52,53]. Therefore, incorporating alternative flours—non-wheat flours—into dietary practices presents substantial opportunities for advancing baked products designed to fulfill diverse nutritional requirements, including those of individuals managing obesity.

Furthermore, essential minerals such as calcium and potassium demonstrated marked increases of approximately 70–85% when compared to the control sample. Samples with higher SMBF also exhibited a notable rise in iron content, reaching levels of 52 to 55 mg 100 g^−1^. Pasha et al. [42] reported a significantly lower iron content in biscuits formulated with 15–25% of mung bean flour, ranging from 7 to 8.5 mg 100 g^−1^. However, this corresponds to an increase of 21–39% compared to control samples. Another study found that incorporating mung bean flour at 60% in biscuit production led to a twofold enhancement in iron content [17]. In contrast, our findings indicated a more modest increase in iron content, estimated at 20–25%. This difference can be attributed to the initial concentration of iron in the raw materials employed, which was determined to be 44 mg 100 g^−1^. Despite these considerations, the observed enhancement can be regarded as both effective and satisfactory, as an 18% increase in ash content relative to the initial data was obtained. Nonetheless, it is noteworthy that the presence of iron in a food product does not necessarily indicate an increased uptake of iron, as several factors can influence its bioavailability and absorption in the body. These factors include the form of iron (heme vs. non-heme), the presence of other dietary components that can enhance or inhibit absorption (such as vitamin C or phytates), and individual variations in digestive health and nutritional status [54]. Furthermore, the prepared Kleija-like biscuits further demonstrated enhanced levels of bioactive compounds, including phenolics, flavonoids, and carotenoids. These bioactive compounds are widely recognized for their significant antioxidant properties, contributing to enhanced health-promoting qualities of food products [19,55]. Research has indicated considerable enhancements in total phenolics, total flavonoids, and antioxidant levels in mung beans due to the sprouting process, with increases documented from 150% to over 200% following a sprouting period of 24–120 h [18,19,56]. In the current work, the incorporation of SMBF at a concentration of 40% resulted in a 16% increase in total phenolic content compared to the control sample. Additionally, total flavonoids and carotenoids exhibited a substantial enhancement, with increases exceeding 50%. Subsequently, significant improvements in antioxidant activity were observed in the formulated samples, with increases ranging from 20% to 45% compared to the control sample. The presence of specific phenolic acids in the formulated biscuits was further evaluated. A total of nine distinct phenolic compounds were identified in the formulated samples, with catechol present at the highest concentration, exceeding 12,000 mg kg^−1^. Moderate levels of other phenolic acids, including benzoic, ferulic, *O*-coumaric, and gallic acids, were also quantified. It is noteworthy that these quantified levels of phenolic acids were exclusively observed in samples with the highest SMBF substitution level (40%). Five distinct flavonoids were also identified in the formulated samples, with significantly elevated concentrations of kaempferol, apigenin, and myricetin. Studies addressing the enhancement of specific phenolics or flavonoids in biscuits formulated with SBMF are currently limited, not to mention the lack of studies even for those made with unsprouted flour. Nonetheless, the SBMF utilized in the current study exhibited a wide range of phenolic and flavonoid compounds, including ferulic acid, caffeic acid, chlorogenic acid, kaempferol, and myricetin. In contrast, various cultivars of unsprouted mung beans exhibited markedly lower levels of these bioactive compounds [57,58]. For instance, Meenu et al. [58] reported approximately 28 mg kg^−1^ of myricetin and 19 mg kg^−1^ of kaempferol, while the current study identified significantly higher levels, estimating myricetin levels at 113 mg kg^−1^ and kaempferol levels at 280 mg kg^−1^. Notably, such compounds have not been identified in commercially available biscuit products [55]. In addition, the control sample analyzed in the current study exhibited significantly lower levels of phytonutrients compared to the formulated samples. These findings underscore the importance of integrating SMBF flour into baked products, as it enhances its phytonutrient content and consequently contributes to improved nutritional quality.

From another perspective, sensory evaluation is a critical component in assessing the quality and acceptability of food products, as it encompasses the analysis of sensory attributes such as appearance, texture, taste, aroma, and overall acceptability [55]. In the current work, the inclusion of SMBF in the biscuits demonstrated a considerable impact on the sensory attributes of the final product. The taste evaluation indicated that samples with higher levels of SMBF content received notably lower preference scores. Specifically, the incorporation of 40% SMBF resulted in the least favorable taste scores among the evaluated samples. Flavor plays an essential role in the overall appeal of food products. This observation suggests that while incorporating SMBF can enhance nutritional content, it may negatively impact sensory attributes if not carefully balanced. It is also noteworthy that biscuits containing a higher proportion of SMBF (40%) showed a decline in texture quality. The texture of biscuits is typically characterized by a harmonious balance of crispiness, contributing to their overall sensory appeal. Crispiness refers to the sound and structural integrity exhibited when a biscuit is broken or chewed, particularly during the initial bites. This sensory attribute significantly affects the product’s overall quality and consumer acceptance [17]. Furthermore, the analysis of visual color conducted by the panelists revealed less favorable scores for biscuits containing 40% SMBF. These results were accompanied by significant differences observed in the colorimeter assay. It was observed that lighter colors were associated with increased concentrations of SMBF. Notably, the lightness in color observed in the current work was accompanied by a decreased browning index when compared to the control biscuits. The darker brownish hue observed in wheat-based baked products is primarily attributed to the Maillard reaction, a complex interplay between sugars and amino acids. The degree of browning can vary significantly based on several factors, including temperature, time, and moisture content during baking. Additionally, the wheat concentration can notably influence the extent of the Maillard reaction. Consequently, a lower concentration of whole wheat flour is associated with a diminished browning index, resulting in a lighter appearance of the final product [59]. Furthermore, this chemical reaction not only contributes to the distinctive browning of the final product but also plays a significant role in developing its flavor and aroma. The formulated biscuits exhibit a pleasant aroma, with the exception of those containing 40% SMBF. The distinctive aroma of biscuits also arises from the volatilization of particular compounds and the complex chemical interactions among the ingredients utilized in the recipe [17]. Overall, incorporating SMBF presents a potential opportunity for developing baked products. Future research should focus on optimizing the levels of SMBF and exploring potential combinations with other ingredients to enhance both flavor and aroma, ensuring that these innovative formulations align with consumer expectations for health and taste.

## 5. Conclusions

The current study demonstrated the potential of utilizing sprouted mung bean flour in the production of Kleija-like biscuits. The findings revealed a significant enhancement in protein quality, as well as increases in fiber and mineral content. Additionally, this study showed a noteworthy presence of phytonutrients, including phenolics, flavonoids, and carotenoids. Additionally, the antioxidant potential was significantly enhanced, emphasizing the capacity of sprouted mung bean flour to serve as a nutrient-dense ingredient in food products designed to improve dietary intake. Sensory evaluation results suggested promising organoleptic attributes; however, it is crucial to maintain a judicious ratio of sprouted mung bean flour to preserve sensory characteristics. It is recommended that the incorporation of sprouted mung beans in future production endeavors be limited to less than 40%. Subsequent studies could explore the feasibility and effects of utilizing a 30–35% sprouted mung bean concentration to optimize favorable overall results.

## Figures and Tables

**Table 1:** Ingredients of Kleija-like biscuit incorporated with SMBF.

Ingredients	Kleija-Like Biscuit Formulas (g)				
	F0	F10	F20	F30	F40
Whole wheat flour	350	315	280	235	210
SMBF	0	35	70	115	140
Oat	50	50	50	50	50
Cow’s butter	250	250	250	250	250
Sucrose fine powder	125	125	125	125	125
Whipped fresh egg	35	35	35	35	35
Spices mix *	16	16	16	16	16
Edible salt	1	1	1	1	1
Citric acid	0.25	0.25	0.25	0.25	0.25

SMBF: sprouted mung bean flour; * Spice mix: 42 g ground cinnamon, 27 g ground cardamom, 18 g ground black lemon.

**Table 2:** Chemical composition of Kleija-like biscuit incorporated with SMBF (mean ± SE), *n* = 6.

Parameter *	Kleija-Like Biscuit Formulas g 100 g^−1^ **				
	F0	F10	F20	F30	F40
Moisture	0.73 ± 0.01 ^d^	1.06 ± 0.08 ^c^	1.35 ± 0.00 ^b^	1.61 ± 0.01 ^a^	1.24 ± 0.11 ^bc^
Crude protein	9.81 ± 0.08 ^e^	9.68 ± 0.06 ^d^	9.92 ± 0.14 ^c^	10.59 ± 0.16 ^b^	11.49 ± 0.01 ^a^
Fat	30.92 ± 0.10 ^a^	30.79 ± 0.28 ^a^	29.88 ± 0.19 ^b^	31.03 ± 0.22 ^a^	29.89 ± 0.25 ^b^
Ash	1.09 ± 0.01 ^d^	1.18 ± 0.02 ^b^	1.13 ± 0.01 ^c^	1.26 ± 0.00 ^a^	1.29 ± 0.00 ^a^
Available carbohydrate	54.95 ± 0.14 ^a^	54.03 ± 0.12 ^b^	53.58 ± 0.06 ^b^	51.46 ± 0.06 ^d^	52.14 ± 0.20 ^c^
Total carbohydrate	57.45 ± 0.07 ^a^	57.30 ± 0.13 ^b^	57.73 ± 0.05 ^b^	55.51 ± 0.03 ^d^	56.09 ± 0.15 ^c^
Crude fiber	4.50 ± 0.07 ^a^	4.27 ± 0.07 ^b^	4.15 ± 0.07 ^bc^	4.05 ± 0.07 ^bc^	3.95 ± 0.07 ^c^
β-Glucan (mg 100 g^−1^)	255.07 ± 7.17 ^a^	267.83 ± 7.53 ^ab^	293.34 ± 8.25 ^b^	306.09 ± 8.61 ^bc^	325.22 ± 9.14 ^c^
Energy (Kcal 100 g^−1^)	529.33 ± 0.44 ^a^	527.94 ± 1.97 ^a^	527.46 ± 1.31 ^a^	523.55 ± 1.57 ^b^	522.85 ± 1.17 ^b^

SMBF: sprouted mung bean flour; *: data were calculated on wet base; **: Kleija-Like Biscuit Formulas (see Table 1); ^a,b,c,d,e^: means sharing the same superscript letters within the same row are not statistically significant (*p* > 0.05).

**Table 3:** Mineral Content of Kleija-like biscuit incorporated with SMBF (mean ± SE), *n* = 6.

Elements (mg 100 g^−1^)	Kleija-Like Biscuit Formulas **				
	F0	F10	F20	F30	F40
Calcium	66.82 ± 0.84 ^e^	71.55 ± 0.9 ^d^	76.27 ± 0.95 ^c^	82.35 ± 1.03 ^b^	85.73 ± 1.07 ^a^
Sodium	14.58 ± 0.18 ^e^	17.51 ± 0.22 ^d^	20.44 ± 0.26 ^c^	24.21 ± 0.30 ^b^	26.3 ± 0.33 ^a^
Potassium	271.19 ± 3.39 ^e^	328.41 ± 4.11 ^d^	385.63 ± 4.83 ^c^	459.2 ± 5.75 ^b^	500.07 ± 6.26 ^a^
Phosphorus	233.14 ± 2.92 ^e^	242.8 ± 3.04 ^d^	252.46 ± 3.16 ^c^	264.87 ± 3.32 ^b^	271.77 ± 3.4 ^a^
Magnesium	87.21 ± 1.09 ^e^	91.01 ± 1.14 ^d^	94.81 ± 1.19 ^c^	99.7 ± 1.25 ^b^	102.41 ± 1.28 ^a^
Manganese	3.14 ± 0.04 ^a^	3.05 ± 0.04 ^b^	2.97 ± 0.04 ^b^	2.86 ± 0.04 ^c^	2.79 ± 0.03 ^c^
Iron	43.75 ± 0.55 ^e^	46.47 ± 0.58 ^d^	49.19 ± 0.62 ^c^	52.69 ± 0.66 ^b^	54.64 ± 0.68 ^a^
Zinc	2.18 ± 0.03 ^c^	2.23 ± 0.03 ^bc^	2.27 ± 0.03 ^ab^	2.33 ± 0.03 ^ab^	2.36 ± 0.03 ^a^
Copper	0.29 ± 0.01 ^e^	0.35 ± 0.01 ^d^	0.41 ± 0.01 ^c^	0.49 ± 0.02 ^b^	0.54 ± 0.02 ^a^
Selenium (µg 100 g^−1^)	16.13 ± 0.2 ^a^	15.23 ± 0.19 ^b^	14.33 ± 0.18 ^c^	13.16 ± 0.16 ^d^	12.52 ± 0.16 ^e^

SMBF: sprouted mung bean flour; **: Kleija-Like Biscuit Formulas (see Table 1); ^a,b,c,d,e^: means sharing the same superscript letters within the same row are not statistically significant (*p* > 0.05).

**Table 4:** Total phenolic content, total flavonoid content, total carotenoids, and relative antioxidant capacity of Kleija-like biscuit incorporated with SMBF (mean ± SE), *n* = 6.

Items *	Kleija-Like Biscuit Formulas **				
	F0	F10	F20	F30	F40
TPC (mg GAE 100 g^−1^)	406.27 ± 7.33 ^c^	415.94 ± 8.06 ^bc^	423.81 ± 5.79 ^bc^	433.02 ± 8.00 ^b^	472.07 ± 1.83 ^a^
TF (mg QE 100 g^−1^)	129.66 ± 0.74 ^e^	166.57 ± 1.45 ^d^	179.89 ± 2.69 ^c^	200.2 ± 2.27 ^b^	212.63 ± 4.30 ^a^
TC (mg GAE 100 g^−1^)	22.02 ± 1.11 ^e^	28.3 ± 1.32 ^d^	30.6 ± 1.05 ^c^	34.0 ± 1.04 ^b^	36.2 ± 0.87 ^a^
DPPH-RSA (mmol of TE 100 g^−1^)	388.97 ± 2.22 ^d^	483.05 ± 4.20 ^c^	503.69 ± 7.54 ^c^	540.53 ± 6.12 ^b^	563.47 ± 11.39 ^a^
ABTS-RSA (mmol of TE 100 g^−1^)	435.65 ± 2.49 ^d^	541.02 ± 4.71 ^c^	564.13 ± 8.45 ^c^	605.39 ± 6.85 ^b^	631.08 ± 12.75 ^a^

SMBF: sprouted mung bean flour; TPC: total phenolic content; TF: total flavonoid; TC: total carotenoids; GAE: gallic acid equivalent; QE: quercetin equivalent; DPPH-RSA: DPPH radical scavenging activity; ABTS-RSA: ABTS radical scavenging activity; TE: Trolox equivalent; *: data were calculated on wet base; **: Kleija-Like Biscuit Formulas (see Table 1); ^a,b,c,d,e^: means sharing same superscript letters within the same row are not statistically significant (*p* > 0.05).

**Table 5:** Phenolics fractionation (mg kg^−1^) of Kleija-like biscuit incorporated with SMBF.

Item	No.	Compounds	SMBF	Kleija-Like Biscuit Formulas **		
				F0	F20	F40
Phenolics	1	Pyrogallol	-	-	-	-
	2	Catechol	-	4879.813	7231.983	12,413.984
	3	*p*-Hydroxybenzoic acid	-	57.388	66.187	95.514
	4	Caffeic acid	78.206	-	-	-
	5	Chlorogenic acid	21.336	-	-	-
	6	Cinnamic acid	-	-	-	-
	7	Ellagic acid	76.492	-	-	-
	8	Vanillic acid	-	640.654	1044.188	398.206
	9	Ferulic acid	251.680	-	-	50.015
	10	Gallic acid	-	-	-	102.706
	11	*O*-coumaric acid	8.687		-	90.836
	12	*p*-coumaric acid	2.439	12.827	-	-
	13	Benzoic acid	159.033	-	-	18.974
	14	Rosmarinic acid	-	-	-	-
	15	Syringic acid	76.492	36.373	-	-
Flavonoids	1	Catechin	-	-	-	-
	2	Kaempferol	284.106	-	18.717	334.866
	3	Myricetin	113.421	-	111.984	125.257
	4	Quercetin	54.054	272.942	21.364	33.056
	5	Rutin	95.051	25.751	-	-
	6	Resveratrol	37.224	-	-	-
	7	Naringenin	279.790	-	-	25.487
	8	Apigenin	-	-	21.077	287.371

SMBF: sprouted mung bean flour; **: Kleija-like biscuit formulas (see Table 1); presented data are mean of duplicate analyses.

**Table 6:** In vitro hydrolysis index (HI), glycemic index (GI), and glycemic load (GL) of Kleija-like biscuit incorporated with SMBF (mean ± SE), *n* = 6.

Formulations **	HI	GI	GL
F0	0.63 ± 0.03 ^a^	40.06 ± 0.04 ^a^	22.01 ± 0.13 ^a^
F10	0.63 ± 0.01 ^a^	40.06 ± 0.01 ^a^	21.64 ± 0.15 ^b^
F20	0.59 ± 0.02 ^ab^	40.03 ± 0.01 ^a^	21.57 ± 0.09 ^b^
F30	0.56 ± 0.01 ^ab^	40.02 ± 0.03 ^ab^	20.59 ± 0.06 ^c^
F40	0.53 ± 0.03 ^bc^	40.00 ± 0.02 ^b^	20.86 ± 0.19 ^c^
SMBF	0.56 ± 0.02	40.02 ± 0.03	21.68 ± 0.11

SMBF: sprouted mung bean flour; **: see Table 1; ^a,b,c^: means sharing the same superscript letters within the same column are not statistically significant (*p* > 0.05).

**Table 7:** Amino acid composition (mg g^−1^ protein) of Kleija-like biscuit incorporated with SMBF.

Amino Acids	Kleija-Like Biscuit Formulas **				
	F0	F10	F20	F30	F40
	Essential Amino acids				
Lysine	26.92	28.79	37.27	41.24	41.52
Threonine	32.45	32.21	33.51	33.21	33.25
Valine	44.29	46.24	50.11	49.67	45.28
Methionine	23.66	22.76	22.61	21.63	21.42
Isoleucine	24.20	26.71	30.35	29.95	35.13
Leucine	65.34	67.23	71.29	71.87	72.80
Phenylalanine	58.07	55.80	61.00	61.91	61.24
Histidine	40.27	38.45	41.14	41.91	48.19
Cystine	28.00	26.60	26.68	25.74	26.21
	Non-Essential Amino acids				
Arginine	41.68	44.79	51.23	56.27	56.27
Aspartic	63.17	69.11	80.25	91.10	88.58
Serine	53.73	52.79	53.88	52.92	52.23
Glutamic	300.55	295.96	286.38	271.96	277.94
Proline	86.94	83.65	42.57	39.71	29.40
Glycine	45.91	43.75	44.30	43.73	43.49
Alanine	43.53	43.33	44.81	45.65	45.56
Tyrosine	21.27	21.82	22.61	21.53	21.51
Total essential amino acids	343.21	344.80	373.97	377.13	385.03
Total non-essential amino acids	656.79	655.20	626.03	622.87	614.97
EAA/TAA ratio	0.34	0.34	0.37	0.38	0.39

SMBF: sprouted mung bean flour; **: Kleija-like biscuit formulas (see Table 1); presented data are mean of duplicate analyses.

**Table 8:** Percentage of amino acids calculated biological efficiency, essential amino acid index, estimated protein efficiency ratio, and requirement index of different age groups.

	Kleija-Like Biscuit Formulas **				
Parameters	F0	F10	F20	F30	F40
Total BCAAs (mg g^−1^ protein)	133.83	140.18	151.75	151.48	153.20
Total Aromatic AAs (mg g^−1^ protein)	79.34	77.63	83.61	83.44	82.75
Total Conditional AAs (mg g^−1^ protein)	532.18	525.62	483.35	468.13	463.55
Total Basic AAs (mg g^−1^ protein)	108.87	112.02	129.65	139.43	145.97
Total Acidic AAs (mg g^−1^ protein)	363.73	365.06	366.64	363.06	366.52
Total Hydrophobic AAs (mg g^−1^ protein)	346.03	345.73	322.74	320.38	310.82
Total Polar AAs (mg g^−1^ protein)	499.19	498.49	503.31	496.46	499.72
BV	45.39	43.67	55.73	55.06	55.35
EAAI	51.63	52.39	57.22	57.61	59.25
Requirement index (Infants)	110.62	112.23	122.57	123.42	126.93
Requirement index (Preschool child)	120.14	121.90	133.13	134.04	137.86
Requirement index (Schoolchild)	131.47	133.39	145.68	146.68	150.85
Requirement index (Adult)	138.26	140.28	153.20	154.26	158.64

**: Kleija-like biscuit formulas (see Table 1); AAs: amino acids; BCAAs: branch-chain amino acids; BV: biological value; EAAI: essential amino acid index.

**Table 9:** Amylograph properties of composite flour with SMBF utilized in producing Kleija-like biscuits.

Items	Kleija-Like Biscuit Formulas **				
	F0	F10	F20	F30	F40
Gelatinization temperature (°C)	60.9	63.0	63.3	64.0	64.6
Peak viscosity temperature (°C)	87.4	78.5	76.1	75.5	74.9
Peak viscosity (AU)	788	348	279	195	168

SMBF: sprouted mung bean flour; **: Kleija-Like Biscuit Formulas (see Table 1); AU: Amylograph units.

**Table 10:** Instrumental and visual color analysis of Kleija-like biscuit incorporated with SMBF (mean ± SE), *n* = 6.

Visual Color Measurements	Kleija-Like Biscuit Formulas **				
	F0	F10	F20	F30	F40
L*	57.79 ± 0.19 ^d^	58.39 ± 0.02 ^c^	61.12 ± 0.15 ^b^	61.14 ± 0.19 ^b^	63.26 ± 0.02 ^a^
a*	7.37 ± 0.08 ^a^	7.34 ± 0.04 ^a^	6.37 ± 0.01 ^b^	5.09 ± 0.04 ^c^	3.37 ± 0.24 ^d^
b*	30.16 ± 0.03 ^a^	29.74 ± 0.08 ^a^	28.39 ± 0.05 ^b^	28.15 ± 0.11 ^b^	27.61 ± 0.57 ^b^
C	31.05 ± 0.02 ^a^	30.64 ± 0.05 ^a^	29.09 ± 0.05 ^b^	28.61 ± 0.11 ^bc^	27.81 ± 0.58 ^c^
*b*/*a*	4.09 ± 0.05 ^c^	4.06 ± 0.03 ^c^	4.45 ± 0.01 ^c^	5.53 ± 0.04 ^b^	8.28 ± 0.58 ^a^
H°	256.3 ± 00.16 ^c^	256.18 ± 0.09 ^c^	257.38 ± 0.02 ^b^	259.79 ± 0.08 ^a^	83.09 ± 0.45 ^d^
BI	80.59 ± 0.41 ^a^	78.09 ± 0.17 ^a^	68.37 ± 0.07 ^b^	66.04 ± 0.52 ^b^	59.64 ± 1.64 ^c^
∆E	0.00 ± 0.00 ^e^	1.09 ± 0.02 ^d^	4.25 ± 0.11 ^c^	4.82 ± 0.19 ^b^	7.58 ± 0.25 ^a^

SMBF: sprouted mung bean flour; L*: lightness; a*: redness; b*: yellowness; *b*/*a*: ratio of the yellow to the red components; C: chroma value; H°: hue angle; BI: browning index; ∆E: color changes. L*, a*, and b* were obtained directly from the Hunter instrument, while C, H°, BI, and ∆E were calculated according to the formulated equation presented in the Materials and Methods (Section 2.6). **: Kleija-Like Biscuit Formulas (see Table 1); ^a,b,c,d,e^: means sharing the same superscript letters within the same row are not statistically significant (*p* > 0.05).

**Table 11:** Sensory characteristics of Kleija-like biscuit incorporated with SMBF (mean ± SE), *n* = 6.

Properties	Kleija-Like Biscuit Formulas **				
	F0	F10	F20	F30	F40
Appearance	19.73 ± 0.15 ^a^	19.13 ± 0.52 ^a^	18.73 ± 0.45 ^a^	19.27 ± 0.42 ^a^	18.80 ± 0.56 ^a^
Taste	18.60 ± 0.62 ^a^	19.20 ± 0.30 ^a^	17.87 ± 0.45 ^a^	17.53 ± 0.70 ^ab^	16.00 ± 0.72 ^b^
Aroma	14.73 ± 0.15 ^a^	14.13 ± 0.29 ^ab^	13.87 ± 0.38 ^b^	14.40 ± 0.25 ^ab^	13.93 ± 0.33 ^ab^
External color	14.93 ± 0.07 ^a^	14.47 ± 0.40 ^a^	14.53 ± 0.17 ^a^	14.60 ± 0.16 ^a^	14.60 ± 0.13 ^a^
Internal color	14.47 ± 0.22 ^a^	14.20 ± 0.37 ^a^	13.87 ± 0.27 ^a^	14.20 ± 0.24 ^a^	14.53 ± 0.22 ^a^
Texture	14.07 ± 0.44 ^ab^	14.00 ± 0.43 ^ab^	14.47 ± 0.19 ^a^	14.47 ± 0.17 ^a^	13.20 ± 0.45 ^b^
Overall acceptability	96.53 ± 1.23 ^a^	95.13 ± 2.11 ^ab^	93.33 ± 1.33 ^ab^	94.47 ± 1.21 ^ab^	91.07 ± 1.30 ^b^

SMBF: sprouted mung bean flour; **: Kleija-Like Biscuit Formulas (see Table 1); ^a,b^: means sharing the same superscript letters within the same row are not statistically significant (*p* > 0.05).

## Data Availability

The original contributions presented in the study are included in the article. Further inquiries can be directed to the corresponding author.
